# Can digitalization and low-carbonization progress in harmony? Evidence from Chinese cities

**DOI:** 10.1371/journal.pone.0292405

**Published:** 2023-10-17

**Authors:** Siliang Guo

**Affiliations:** 1 School of Economics and Management, Qilu Normal University, Jinan, Shandong, China; 2 School of Economics and Management, Nanjing University of Aeronautics and Astronautics, Nanjing, Jiangsu, China; Qufu Normal University, CHINA

## Abstract

Achieving high-quality development of the city requires actively promoting coordinated digitalization and low-carbon development. Previous studies have focused on the unidirectional impact of urban digitalization on low-carbonization and there is a lack of research on their interactions. This study uses the generalized spatial three-stage least squares method and the spatial simultaneous equation to investigate the endogenous interactions between urban digitalization and low-carbonization. The properties of the spatiotemporal evolution are then examined using linked coordination degree models, kernel density, and spatial statistical approaches. Finally, using the spatial panel metering model, this study empirically investigates the motivations behind the synergistic advancement of digitalization and low-carbonization. The results show that: (1) There is an endogenous interaction between urban digitalization and low-carbonization and that this interaction pattern is closely linked to geographical proximity. (2) In general, both urban digitalization and low-carbonization have a positive spatial impact and a negative spatial interaction, and their coordination levels have a significant spatial impact. (3) Throughout the research period, the coordination degree of urban digitalization and low carbonization continued to increase, showing a positive spatial correlation and a balanced development trend. (4) Economic development, industrial structure, and human capital accumulation are vital internal drivers of the synergistic advancement of urban digitalization and low carbonization. Government capacities and technological innovations are key external factors that contribute to the synergistic advancement of urban digitalization and low-carbonization. Overall, the paper is essential not only to deepen understanding of the relationship between urban digitalization and low-carbonization but also to formulate policies for their coordinated development.

## Introduction

Digitalization and low-carbon development have emerged as the two main directions for future global development [1–3]. With the development of digital technologies such as the Internet, big data, artificial intelligence, and other technologies, countries are competing with the development of digital strategies and the introduction of policies to promote digitalization. Digitalization has emerged as a strategic alternative for countries throughout the world seeking to capitalize on the current prospects presented by the new wave of technological revolutions and industrial transformations [4]. China has created the "Digital China" development strategy and accords the growth of the digital economy a great priority. Meanwhile, With the increasing pressure to reduce carbon emissions due to climate change, the process of reducing carbon emissions and promoting low-carbon development of cities through urban digital construction has become an issue of deep concern in the theoretical community and the focus of policy makers [5]. According to the International Exponential Climate Action Roadmap, the effort could help cut global carbon emissions by up to 15% through digital solutions in the energy, manufacturing, and other sectors by 2020 [6]. By 2030, carbon emissions must be reduced by 50%; this decrease represents one-third of that amount. China has continuously outlined the path of carbon neutrality in its national policies for the growth of the digital marketplace, taking factors like digital infrastructure, new energy, innovation, and industrial digitalization as key pillars for the strategy. In this context, it is of great theoretical and practical value to explore the relationship between urban digitalization and low-carbonization.

The boost and spatial spillover effects of digitalization on low-carbon development have been validated by a number of theoretical and empirical studies. Not only does digitalization have a direct role to play in promoting low-carbon development, but it can also affect it through technological advances, industrial restructuring and digital regulation by governments [7–9]. But will low-carbonization have a backfiring effect on urban digitalization? Are there spatial interplay spillover effects between urban digitalization and low-carbonization? What are the spatio-temporal evolution characteristics and key drivers for the interactive and collaborative development of urban digitalization and low-carbonization? These questions need to be explained and tested. The main aim of this paper is to attempt to answer the above questions by combining theory and demonstration, and to provide empirical evidence for the harmonious co-development of urban digitalization and low-carbonization. Therefore, based on data from 282 Chinese cities from 2011 to 2019, this paper first theoretically combed the interaction mechanisms and influence factors of the co-promotion of digitalization and low-carbonization, and then empirically examined the endogenous interplay of the two by constructing spatially simultaneous equations. At the same time, a coupled coordination evaluation model is constructed to assess the degree of collaboration between urban digitalization and low-carbonization using 3D kernel density and spatial statistical analysis, and to reveal its spatial distribution pattern and spatio-temporal evolution characteristics. Finally, based on the spatial metrology model, we also point out the main forces behind the collaborative promotion of urban digitalization and low-carbonization.

This paper’s marginal contribution is as follows: (1) Extend research on the relationship between digitalization and low-carbonization. Previous research has solely looked at the one-way influence of digitalization on low-carbonization, neglecting the spatial interplay between the two. In this study, we propose a theoretical method for coordinating the interplay of digitalization and low-carbonization based on the orientation of energy conservation and efficiency improvement, as well as the spatial scale of cities at the prefecture level and higher. We experimentally evaluate the bidirectional connection between the two using the spatial simultaneous equation and the generalized spatial three-stage least square (GS3SLS) approach, which allows for a more thorough understanding and assessment of the cooperative development link between urban digitalization and low-carbonization. (2) Gain a better knowledge of the regularity with which urban digitalization and low-carbonization are coordinated. The synergy degree of digitalization and low-carbonization in Chinese cities was fully examined using system theory. Simultaneously, the spatiotemporal evolution traits of the synergy degree are investigated, providing the coupling coordination development level and spatial correlation effect of digitalization and low-carbonization in different cities, and providing a decision-making basis for cities to formulate effective development policies and realize high-quality development by better-utilizing digitalization and low-carbonization. (3) To add to the empirical information on the elements impacting low-carbon urban development from a synergistic standpoint. We conducted an empirical study in this paper using a spatial panel model, which captures the key influencing factors that influence the cooperative promotion of digitalization and low-carbon advancement in urban areas and can help establish regulations for the cooperative development of digitalization and low-carbonization.

The rest of the paper is structured as follows. Section 2 examines the pertinent literature. Section 3 develops the theoretical foundation of this study. The model, variables, and data sources utilized in this work are described in Section 4. Section 5 shows the empirical findings. The discussion is presented in Section 6. The conclusions are presented in Section 7.

## Literature review

The prior literature has concentrated on the influence of digitalization on low-carbonization but has not achieved a clear conclusion on the two’s causal link.

The majority of research have suggested that digitalization is beneficial to carbon reduction, and related studies have mostly focused on the direct, indirect, and geographical spillover impacts of digitalization on carbon reduction. (1) Digitalization has a direct impact on carbon reduction. First, digitalization can help accomplish sustainable development goals by building digital infrastructure [10]. Traditional facilities can be altered and improved to build new infrastructure as digital technology advances. Smart manufacturing workshops, for example, can forecast and regulate carbon emissions using digital information technology [11]. Investment in smart hospitals, smart energy, smart transportation, and other digital infrastructure can effectively reduce carbon emissions [12–14]. Building a "smart city" using digital technology, as well as fully developing and utilizing renewable energy, are both viable options for reducing carbon emissions [15]. Second, efficient integration of ICT and traditional sectors promote carbon reduction. Traditional industries can map out improved manufacturing schemes by gathering and analyzing information, and continually cutting emission levels per unit of output, with the use of big data and machine learning [16]. Furthermore, because it is based on information technology, digitalization might give additional impetus for smart environmental management [17]. In addition to having the potential to significantly improve the ecological environment, widespread adoption and utilization of digital technologies in energy consumption and environmental protection can help address the issues of decreasing ecological carrying capacity and scarceness [18, 19]. According to Asongu [20], the development of the Internet in Africa could increase trade accessibility and lower carbon emissions. Information technology, according to Danish et al. [21] and Tsaurai and Chimbo [22], has expedited financial development and dramatically decreased carbon emissions in emerging markets. According to Guo et al. [23], the development of China’s smart cities can reduce the amount of carbon dioxide emissions per person and save energy, with a decreased emission affecting roughly 18.42 logistic percent. (2) The indirect impact of digitalization on reducing carbon emissions. In addition to fostering the development of green technologies [24, 25], digitalization can also improve industry structural efficiency and lessen information asymmetry [26]. At the global scale, governments can more effectively manage total carbon emissions with the help of digital technology by better understanding the energy market and price patterns and managing the overall energy supply through pricing and cross-subsidies [27]. An industrial structure characterized by laborious and costly industries can be replaced with one that is dominated by high-tech content and environmental friendliness thanks to digitalization, a new economic transformation driver [7]. In addition, new-generation digital technologies, like artificial intelligence, have distinct application prospects in various industries and can replace labor or capital [28]. These technologies also aid in the process of industrial change and upgrading by speeding up the movement of production factors between industries [29]. According to a micro perspective, digital transformation can be a catalyst for businesses to innovate in green technology, which unquestionably has an impact on emission levels [30]. Traditional methods of working and consuming have changed as a result of the utilization of digital technologies. By reducing investment in brick-and-mortar businesses and dependency on heavy sectors like coal and cement, the rapid expansion of electronic commerce and internet-based platforms has aided in the greening of the economy [31]. Digitalization has the potential to increase the knowledge intensity of production connections and the permeability of data production elements, so facilitating the growth of firms toward intensive production, improving resource allocation efficiency [32], and achieving lower emissions. (3) Digitalization’s spatial spillover implications on carbon reduction. Because of digital technologies’ great geographical correlation and permeability, digitalization could assist in the establishment of coordinated regional carbon reduction patterns via spatial spillover effects [33]. The regional spillover of digital carbon reduction impacts varies between economic circles due to variations in geographical borders [34], resulting in clear spatial heterogeneity [35].

According to some academics, the digital building has a detrimental influence on carbon reduction. While digital technology fosters advancement in society and the economy, rising electricity consumption might result in a considerable rise in carbon emissions [36]. While the efficiency of energy savings and emissions reductions from digitalization continues to improve, processor energy consumption is halving every 1.5 years, and new processor architectures are becoming more energy-efficient and effective [37, 38]. Some researchers have noted that as the ICT sector has grown rapidly, total carbon emissions have also grown exponentially [16, 39, 40]. The advancement of the digital techniques involves the collection, transmission, and processing of vast volumes of data, which consumes considerable amounts of electricity and increases the release of carbon dioxide [41–43]. In addition, due to the relatively short technical life of ICT gear and equipment, the rapid advancement of digital technologies accelerates the updated pace of digital goods, which greatly increases energy consumption [44, 45].

Scholars seldom address how low-carbonization affects digitalization. Few academics have investigated the influence of low-carbonization on digitalization from the standpoint of technical innovation. On the one hand, low-carbonization necessitates greater investment in digital technology innovation. Liu et al. [46] examined the panel vector autoregression (PVAR) model and discovered that carbon emission efficiency might induce a short-term increase in scientific and technological innovation. However, with time, this good influence fades and progressively becomes negative. Low-carbonization, on the other hand, will offer a green atmosphere for the growth of digital technologies. Due to increased innovation costs and more complicated innovation processes in the context of the low-carbon age, where digital technologies are defined by green development friendliness, such technological advancements frequently impose larger demands on inventive knowledge resources [47]. Environmental ethics, environmental regulations, green providers, and strengthening the absorptive capacity of companies relying on low-carbon and environmentally friendly development can exert a significant impact on technological innovation, including digital technology, in order to move toward green development [48]. In addition, digitalization and low-carbonization are both essential drivers of high-quality urban growth in both economy and society and coordinated development of both is critical for regional development. Although some researchers have testified to the link between digitalization and green development from a coupled coordination approach [49], there still exists a scarcity of studies that explicitly investigates the coordinated development of digitalization and low-carbonization.

In short, most scholars are currently focused on empirical tests of the one-way effect of digitalization on low-carbonization, or on coupled coordination relation analysis without interaction relation tests. At the same time, there is a lack of systematic studies of the interplay between urban digitalization and low-carbonization taking into account spatial factors, temporal and spatial characteristics, and drivers, and even less related studies of the spatial interplay between urban digitalization and low-carbonization from the urban level. Thus, the current literature on digitalization and low-carbonization provides a good framework for this study, but there are still some gaps: (1) Existing studies have mainly focused on one-way impact tests between digitalization and low-carbonization, without empirical tests for possible two-way interaction effects between the two. (2) Most of the existing research has focused on the intrinsic effects of digitalization and low-carbonization, and there is a lack of systematic investigation of the geographical spillover effects and spatial interplay effects of the two. There have been analyses based directly on coupled coordination models that lack both empirical tests of interaction effects and consideration of spatial spillover effects. (3) Empirical studies on the systematic analysis of spatial patterns, evolutionary signatures and drivers of digitalization and low-carbonization co-promotion from the perspective of spatial interactions are insufficient and need to be further supplemented. (4) The study sample consists mostly of regional, national, or provincial levels, and the geographical size of the sample was insufficient, particularly given the paucity of empirical data from the urban level.

In view of this, the goal of this paper is to try to remedy the above deficiencies. Specifically, using 282 Chinese cities as a sample, this paper is the first to empirically test whether there is a spatial interaction between urban digitalization and low-carbonization. The spatio-temporal patterns and evolutionary properties of the interactions are revealed based on the presence of urban digitization and low-carbonization spatial interactions. Finally, the driving factors for the harmonious progress of urban digitalization and low-carbonization are explored under the assumption that spatial spillover effects are fully taken into account.

## Theoretical basis

**Fig 1:**
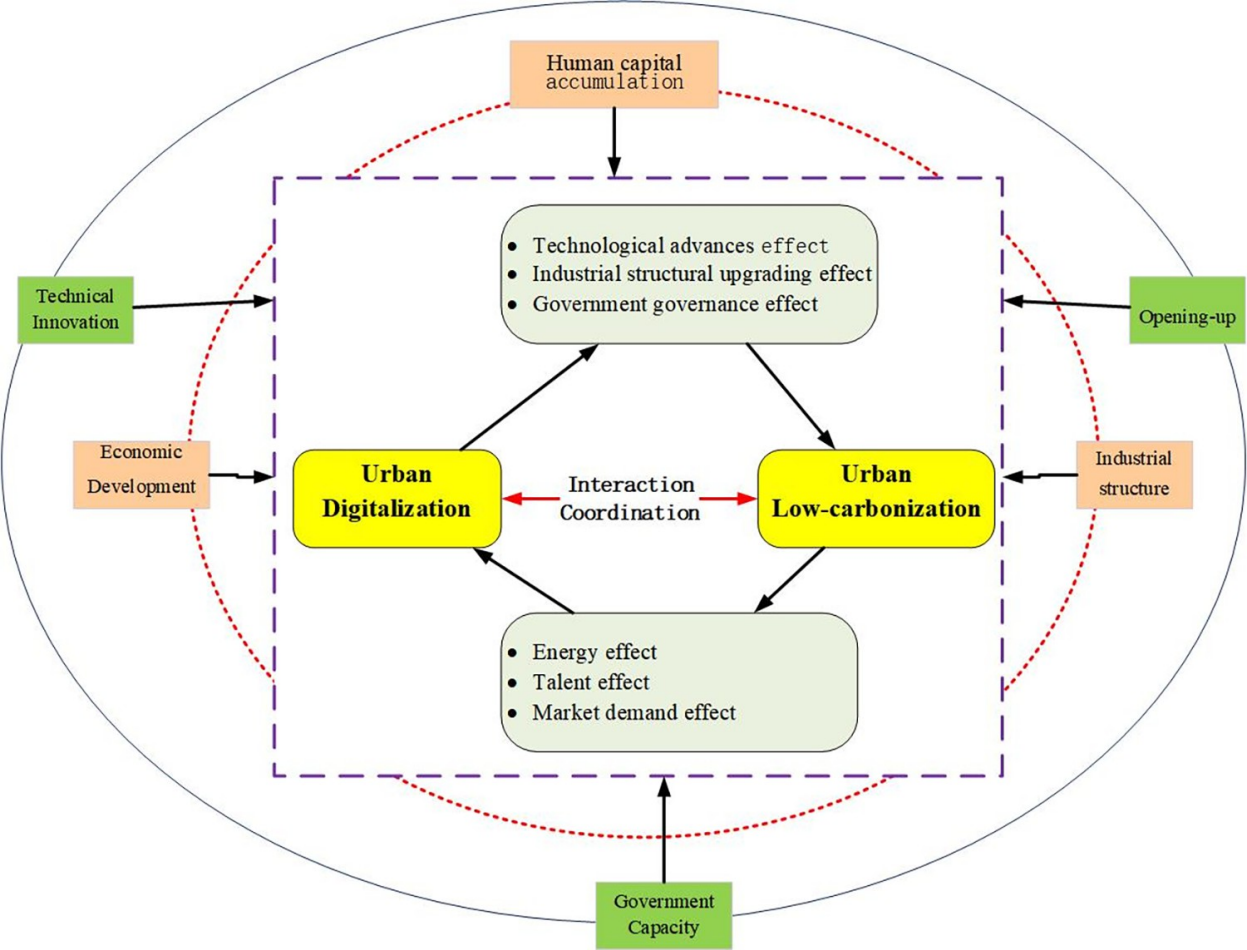
Framework for theoretical analysis.

Fig 1 shows the framework of theoretical analysis in this paper.

### Coordination and interaction between urban digitalization and low-carbonization

#### Urban digitalization promotes low-carbonization

Urban digital construction can promote low-carbon development through technological advances, industrial structural upgrading, and improved government governance.

First, the effect of technological advances. The continuous improvement of urban digitalization has established a solid technological foundation for low-carbonization. At a macro level, the expansion of digitalization can help the advancement of green technology [50]. The adoption and combination of advanced digital technologies including smart grids, intelligent control, energy-saving technologies, and new materials technology can help establish access standards for energy digital technologies and speed up the evolution and advancement of energy-efficient innovations to help eliminate outdated manufacturing capabilities and encourage development that is environmentally friendly and low-carbon [51]. At the micro level, digitalization will expedite the spread of digital low-carbon technologies, hasten the spread of green technology to other industries, and facilitate the digital and low-carbon evolution of conventional firms [52].

Second, the upgrading effect of industrial structure. Low-carbon development requires an advanced industrial structure [53, 54]. Capital can flow into digital-related firms and sectors with high efficiency, tremendous potential, and high returns, guided by market forces. By reallocating capital through financial intermediation and financial markets, more limited resources are invested in regions or sectors with higher productivity and output growth rates, increasing resource efficiency and low-carbon development across societies and regions [55]. On the one hand, the convergence of digitalization and conventional sectors has helped traditional industries’ digital transition [56]. The integration of cloud computing, artificial intelligence, and other future digital innovations into traditional industries has assisted enterprises in improving their smart, flexible, and low-carbon manufacturing and R&D processes, accelerating the upgrading of traditional manufacturing industries, optimizing the allocation efficiency of energy-intensive industries, and reducing enterprise overcapacity and production losses [57, 58]. On the other hand, digitalization has the potential to spur the growth of new digital sectors. The extensive incorporation of digital technologies with luxurious intelligent production, electronic data, and other high-tech sectors has facilitated the rise and growth of new digital industries, reducing industrial development’s reliance on high-carbon energy [7], encouraging the shift of industrial structure to digital and decrease-carbon, lowering energy use and carbon emissions, and promoting the low-carbon development of cities.

Third, the improved impact of government governance. Digitalization can enhance the ability of governments to manage low-carbon policies. Through the integration of data resources and digital technologies, digitalization provides substantial backing for the development of powerful [8] and accurate carbon data service platforms and digital network systems, which significantly reduce the cost of carbon information search, classification and calculation, and effectively improve the government’s information sharing and intelligent management of data resources such as carbon emissions and carbon sinks. Digitalization also effectively promotes the government to discover the "carbon background", carry out "carbon screening", do a good job in "carbon planning", optimize "carbon trading", and promote "carbon emission reduction" [47, 59], significantly enhances the government’s capacity of carbon monitoring and management and low-carbon governance, and thus promoting the low-carbon development of cities.

#### Urban low-carbonization drives digitalization

Low-carbonization is an unavoidable prerequisite for urban digital building, which helps to improve digital construction quality. Through the energy impact, talent effect, and market demand effect, Low-carbonization could promote urban digitalization.

One is the energy effect. As a whole, digitalization uses a lot of energy, especially electricity [60, 61]. It is urgent to intensify energy conservation and carbon emission reduction efforts, improve technology, accelerate innovation, improve energy efficiency, reduce energy consumption, reduce carbon emissions, and steering science and technology in a direction that is conducive to reducing carbon emissions under the dual pressure of economic development transformation and carbon emission reduction, in the construction process of 5G, data centers, industrial Internet, and other forms of high energy consumption.

The second is the talent effect. Low-carbon development promotes digital development through the talent agglomeration effect resulting from the optimization of the ecological environment. Deterioration of the ecological environment leads to increased public health costs and increased risk of net loss of social labor. In addition, people with higher levels of education have higher standards for quality of life [62], which will directly affect the introduction of top talent and the retention of undeveloped technologically advanced talent and indirectly affect the development of human capital and the region’s level of technology [63, 64]. Low-carbon development has improved the city’s ecological environment and quality of life, encouraging residents to strive for higher standards of living [10], enhancing the personal quality of life, and promoting low-carbon consumption, all of which can contribute to a favorable environment and steady inflow of talent for the city’s digital construction.

A low-carbon society is replacing industrial civilization in human society. With its deep integration with the agricultural, industrial, manufacturing, and service sectors, digitalization—which is typical of the contemporary era—will bring about comprehensive economic and social reform [65–67]. The degree of low-carbon application technology must be raised with the aid of low-carbon technological innovation in order to create a modern digital city system that is energy-intensive, effective, inexpensive, intelligent, green, and dependable. Therefore, a wide range of application scenarios and market demand for digital construction will be provided by the strong need for green and low-carbon technological innovation, which will speed up the development of urban digitalization [68].

### Driving factors of coordinated development of urban digitalization and low-carbonization

#### Internal driving factors

Economic development, human capital, and industrial structure are critical internal drivers for coordinating urban digitalization and low-carbonization. Although the complex relationship between economic growth and environmental pollution has been demonstrated by the Kuznets curve of carbon emissions [69]. The intrinsic need for high-quality development, however, can push the shift of urban sustainable and low-carbon growth patterns and also serve as the material basis for the digital building when the level of economic development rises. Accumulation of human capital is a new engine for raising productivity and a crucial component of the concerted push for urban digitalization and low-carbonization. The following are some of the reasons why increasing human capital can help digitalization and low-carbonization work together more effectively. On the one hand, boosting human capital quality can help offset the negative impacts of declining marginal returns on physical capital and CO2 emissions [70] and fulfill the dual aims of growth and reducing emissions. Conversely, the more educated people are, the more aware they are of the serious consequences of ecological degradation and the potential benefits of environmental protection, and the more willing they are to adopt low-carbon behaviors and lifestyles, thereby facilitating low-carbon development in the digital transformation [62]. Carbon emissions can be efficiently reduced by developing industrial structures. Not only is the simplification of industrial structure necessary for carbon reduction, but improving industrial structure is also necessary for carbon reduction [71]. Additionally, industrial structure conversion and upgrades have established the software foundation for the city’s digital building.

#### External driving factors

External factors for the coordinated growth of urban digitalization and low-carbonization include technological innovation, government capability, and openness to the outside world. Technology innovation is a critical driver of coordinated urban digitalization and low-carbon development. On the one hand, the knowledge spillover effect of technical innovation can stimulate technological advancement in associated industries, regions, and micro-enterprises by encouraging and directing upstream and downstream companies to lower carbon intensity [72]. Therefore, low-carbon technical innovation at the firm level can contribute directly to the creation of a low-carbon economy, finally achieving the goals of energy conservation and reducing emissions. On the other hand, digital technology innovation can not only accelerate technology diffusion and increase enterprise productivity and quality, but it can also promote continuous improvement of the digital industrial chain [73], which is critical to enhancing the effectiveness of urban digital transformation. It is difficult for the market mechanism to reach Pareto optimality in the process of urban digitalization and low-carbon development, which necessitates the participation of government agencies. First, the government has incorporated low-carbon concepts into the entire digital construction process through the development of low-carbon city pilot plans and other plans, as well as mobilizing the subjective initiative of various stakeholders in a flexible and effective manner, resulting in the formation of a differentiated low-carbon way to progress according to the features of different regions and industries [74]. Second, government-led digital city construction focuses on improving the clean energy system, low-carbon transportation system, and green pipeline transportation system, lowering the carbon intensity of digitalization, guiding consumers to form low-carbon tastes, and enterprises to choose low-carbon technologies, thereby helping to optimize the energy consumption in the digital construction process [75]. The degree of opening-up of a city directly affects the depth of industrial participation in economic globalization. Industries can participate in economic globalization through foreign direct investment, absorb advanced science and technology and management experience, and obtain knowledge spillovers and innovation [76]. However, the influx of foreign capital can have a crowding-out effect, affecting urban industrial development and hindering urban digitalization and low-carbon development [77].

## Research design

### Indicator’s description

#### Urban digitalization (UD)

The measurement of the level of digitalization is not yet mature, and most researchers have conducted a similar study by referring to informatization measuring metrics. Based on the work of Zhao et al. [78] and Huang et al. [79], this paper develops six metrics for evaluation according to the three dimensions of digitalization foundation, digitalization development, and digitalization benefits, and extensively evaluates the level of urban digitalization after weighting by the entropy value method.

#### Urban low-carbonization (UL)

In this paper, we use the urban carbon emission efficiency index to reflect the level of low-carbonization. Based on the strategy of Shan et al. [80], this paper calculates the CO2 emissions from fossil fuel combustion from top to bottom by multiplying the city’s usage of fuel by the associated carbon transformation factors. Based on Tone and Tsutsui [81], we adopt the ultra-efficient EBM model in this paper for calculating carbon emission efficiency by taking as inputs the region’s fixed capital stock, the number of employees, and total electricity consumption at the end of the year, the gross regional product as the expected output, and CO2 emissions as the unanticipated output.

#### Description of the other variables

In this paper, we add nine control variables to the simultaneous equation model and six key influence factor variables, and two control variables to the spatial econometric model, according to theoretical understanding and existing research experience [78, 79, 82, 83].

Table 1 lists the variables that were employed in the empirical study.

**Table 1:** Indicator’s description.

Level 1 Dimension	Level 2 Dimension	Indicator	Explanation
**Urban digitalization**	Foundation	Internet users per 100 people	
		Proportion of computer service and software employees	
	Development	Total telecom services per capita	
		Total postal services per capita	
	Benefit	Number of mobile phones per 100 people	
		Digital Financial Inclusion Indicator	
**Urban Low-carbonization**	Input	Capital	Fixed capital stock
		Labour force	Number of employees at the end of the year
		Energy	Total electricity consumption
	Output	Desirable Output	Gross domestic product
		Undesirable Output	Carbon dioxide emissions
**Control variables in simultaneous equation models**	Digitalization equation	Human capital (HC)	The product of total population and average years of schooling
		Government Capacity (GC)	Fiscal revenue per capita
		Opening-up (OU)	Proportion of actual utilization of foreign capital to GDP
		Technical Innovation Capability (TI)	Patent applications per 100 people
	Low-carbonization equation	Economic Development (ED)	GDP per capita
		Industrial structure (IS)	Proportion of output value of secondary industry to tertiary industry
		Communal facilities (CF)	Number of cars and trams operated per capita
		Urban size (US)	Built-up area of a city
		Environmental regulation (ER)	PM2.5 concentration
**Variables in spatial econometric model**	Key driver variables	Economic Development (ED)	GDP per capita
		Industrial structure (IS)	Proportion of output value of secondary industry to tertiary industry
		Human capital (HC)	The product of total population and average years of schooling
		Government Capacity (GC)	Fiscal revenue per capita
		Opening-up (OU)	Proportion of actual utilization of foreign capital to GDP
		Technical Innovation Capability (TI)	Patent applications per 100 people
	Control variables	Communal facilities (CF)	Number of cars and trams operated per capita
		Urban size (US)	Built-up area of a city

### Methods

#### Spatial simultaneous equation model

Traditional single equation models have difficulty in providing unbiased and consistent estimation results for interactions between variables. Therefore, based on the framework of simultaneous equations for panels, we model the coordination between UD and UL and reconstruct the UD (1) and UL (2) equations. Meanwhile, considering the possibility of simultaneous bias in the system of simultaneous equations, we follow the approach of Chu et al. [84] and use a three-stage least squares method (3SLS) for estimation. The specific form of the UD and UL equations is as follows.


$${UD}_{it}=\beta_{0}+\beta_{1}{UL}_{it}+\beta_{2}X_{it}+\mu_{i}+\sigma_{t}+\varepsilon_{it}$$ (1)

$${UL}_{it}=\gamma_{0}+\gamma_{1}{UL}_{it}+\gamma_{2}Z_{it}+\xi_{i}+\delta_{t}+\upsilon_{it}$$ (2)

In Eq (1), *t* and *i* stand for the year and city, respectively. The explained variable is UD and the core explained variable is UL. *X*_*it*_ represents control variables. *μ*_*i*_ and *σ*_*t*_ are individual and time effects, respectively. *ε*_*it*_ represents the random errors. In Eq (2), The explained variable is UL and the core explained variable is UD. *Z*_*it*_ represents control variables. *ξ*_*i*_ and *δ*_*t*_ are individual and time effects, respectively. *υ*_*it*_ represents the random errors.

Although the simultaneous equation model takes into account the relationship between endogenous variables, it still neglects the spatial spillover effects of core variables and the resulting bias of missing variables. In the presence of spatial spillovers, A change in an explanatory variable impacts not only the explanatory variable of the localized area but also the explanatory variable of the area around it, causing the local region to suffer as a result [85]. For this purpose, the panel simultaneous equation model is further extended to the panel space simultaneous equation model. In fact, it is not easy to estimate the parameters of a panel simultaneous equation model with spatial effects, which must rely on special estimation techniques for identification. In this paper, the GS3SLS method proposed by Kelejian and Prucha [86] is adopted for estimation, and the spatial lag term of endogenous variables is introduced into the model. At the same time, the correlations among the stochastic disturbance terms in the system of equations are treated accordingly. The specific model is as follows:

$${UD}_{it}=\beta_{0}+\beta_{1}{UL}_{it}+\rho_{1}\sum_{j=1}^{N}W_{ij}{UL}_{jt}+\rho_{2}\sum_{j=1}^{N}W_{ij}{UD}_{jt}+\beta_{2}X_{it}+\mu_{i}+\sigma_{t}+\varepsilon_{it}$$ (3)

$${UL}_{it}=\gamma_{0}+\gamma_{1}{UL}_{it}{+\rho }_{3}\sum_{j=1}^{N}W_{ij}{UL}_{jt}+\rho_{4}\sum_{j=1}^{N}W_{ij}{UD}_{jt}+\gamma_{2}Z_{it}+\xi_{i}+\delta_{t}+\upsilon_{it}$$ (4)

Eq (3) and Eq (4) are based on Eqs (1) and (2) with the addition of the space lag terms of UD and UL. *W* is the space weight matrix. *ρ* is the spatial correlation coefficient of an endogenous explanatory variable to measure the spatial spillover effect of UD and UL.

#### Coupling coordination model

The coupling coordination model can indicate both the degree of connection and the interaction between the systems. We use the coupling coordination model to highlight the intensity of interaction and synergy degree of digitalization and low-carbonization. The model’s particular calculation procedure [87] is as follows:

(1) System coupling degree (C)

$$C=2\sqrt{\frac{UD\times UL}{{\left(UD+UL\right)}^{2}}}$$ (5)

(2) coupling coordination degree (*CD*)

$$CD=\sqrt{C\times (\alpha \times UD+\beta \times UL)}$$ (6)

Where, *α* and *β* are harmonic coefficients, *α*+*β* = 1. In this paper, *UD* is considered to be equally important as *UL*, so *α* = *β* = 0.5.

#### Kernel density method

We use a 3D kernel density curve to characterize the distribution form, location, and scalability of the synergy between urban digitalization and low-carbonization in order to explore the dynamic change in the synergistic advancement of urban digitalization and low-carbonization. The approach of nonparametric kernel density is used to estimate probability density functions. Kernel density estimation methods characterize the distribution form of random variables using continuous density curves, and the predicted results are more consistent than histograms. The formula is as follows [88]:

$$f_{N}(X)=\frac{1}{Nh_{N}}\sum_{i=1}^{N}K(\frac{X_{i}-X}{h_{N}})$$ (7)

Where, *f*_*N*_(*X*) is the kernel density function of random variable X; N represents the number of cities; K is the kernel function of variable X, and adopts Gaussian kernel function; *h*_*N*_ is the bandwidth.

#### Spatial effect model

The global spatial correlation tests refer to examine the attribute values of neighboring area units throughout the region, generally by measuring Moran’s I (GMI), which is measured as in Eq (6) [89]:

$$GMI=\frac{\left(\frac{m}{\gamma_{0}})\times \{\sum_{i=1}^{m}\sum_{j=1}^{n}W_{ij}(D_{i}-\bar{D})(D_{j}-\bar{D})\right\}}{\sum_{i=1}^{m}{\left(D_{i}-\bar{D}\right)}^{2}}$$ (8)

*Where*, m is the number of prefectures and cities, and n is the number of neighboring cities. *D*_*i*_ and *D*_*j*_ represent the coupling coordination degree for cities i and j, respectively. $\bar{D}$ is the mean value of the coupling coordination degree. *γ*_0_ is the element of the weight matrix. *W*_*ij*_ is the spatial weight matrix.

Local spatial correlation tests are used to assess whether there is a clustering effect in the local space of data [90]. In order to analyze the atypical character of localized states, local spatial correlations were examined by the local Moran’s I (LMI), and the significance was expressed using the LISA significance test. The formula for calculating local spatial correlations is as follows [91].


$$LMI=\frac{D_{i}-\bar{D}}{S^{2}}\sum_{j\neq i}W_{ij}(D_{j}-\bar{D})$$ (9)

*Where*, *S*^2^ represents the variance of coupling coordination degree.

#### Panel space measurement model

Neglecting spatial effects can lead to severe missing variable bias. In order to accurately identify important variables determining the extent of coordinated urban digitalization and low-carbonization, and to account for spatial autocorrelations arising from spatial spillovers, the following spatial measurement models were developed:

$${CD}_{it}=\alpha_{0}+\eta {CD}_{it-1}+\rho \sum_{j=1}^{N}W_{i\dot{J}}CD_{it}+\beta X_{it}+\gamma \sum_{j=1}^{N}W_{i\dot{J}}X_{it}+\mu_{i}+\sigma_{t}+\varepsilon_{it}$$ (10)

$$\varepsilon_{it}=\theta \sum_{j=1}^{N}W_{ij}\varepsilon_{jt}+\zeta_{it}$$ (11)

Where, i and t represent the city and time, respectively. *W*_*ij*_ is the spatial weight matrix. *ρ* and *γ* stand for the coefficients of spatial autoregressive and autocorrelation, respectively. *X* represents explanatory variables. *ε*_*it*_ is the spatial errors and *ζ*_*it*_ represents stochastic disturbance. In this study, we built four types of measurement models under unusual conditions, specifically: spatial autoregressive model (SAR: *θ* = 0 and 03b3 *γ* = 0), spatial autocorrelation model (SAC: η = 0 and *θ* = 0), spatial error model (SEM: *ρ* = η = 0 and *γ* = 0) and spatial Dubin model (SDM: *θ* = 0). The study employs the fixed effect model for estimating using the Hausman test, and the estimation technique is MLE.

#### Data sources

Considering the accessibility and reliability of data for the specified variables, we excluded cities with serious data gaps from this work, leaving 282 cities as study subjects. The statistics on digital inclusive finance in this research originate from the Digital Inclusive Finance Index [92] at the prefecture and city levels. The following data are mostly from the China Municipal Statistical Yearbook, the China Municipal Construction Statistical Yearbook, the China Research Statistics Service Platform, and statistical yearbooks and bulletins of cities at and above the prefecture level from 2012 to 2020. The few missing values are linearly interpolated. Meanwhile, non-ratio metrics are treated logarithmically to reduce sample variation. Table 2 shows the descriptive statistics of the variables used in this paper.

**Table 2:** Descriptive statistics.

VARIABLES	N	Mean	Sd	Min	Max
**UD**	2,538	0.0824	0.0663	0.00777	0.780
**UL**	2,538	0.493	0.0676	1.00e-07	1.000
**HC**	2,538	8.076	0.701	5.118	10.28
**GC**	2,538	8.085	0.867	5.791	11.31
**OU**	2,538	0.260	0.274	0	2.990
**TI**	2,538	0.162	0.347	0.0009	4.713
**ED**	2,538	10.70	0.576	8.773	13.06
**IS**	2,538	1.267	0.615	0.193	8.802
**US**	2,538	4.561	0.843	2.565	8.123
**CF**	2,538	3.132	3.804	0.0947	98.68
**ER**	2,538	0.438	0.152	0.136	1.089

## Empirical results

### Test results on the interplay between urban digitalization and low-carbonization

#### Spatial correlation analysis

**Fig 2:**
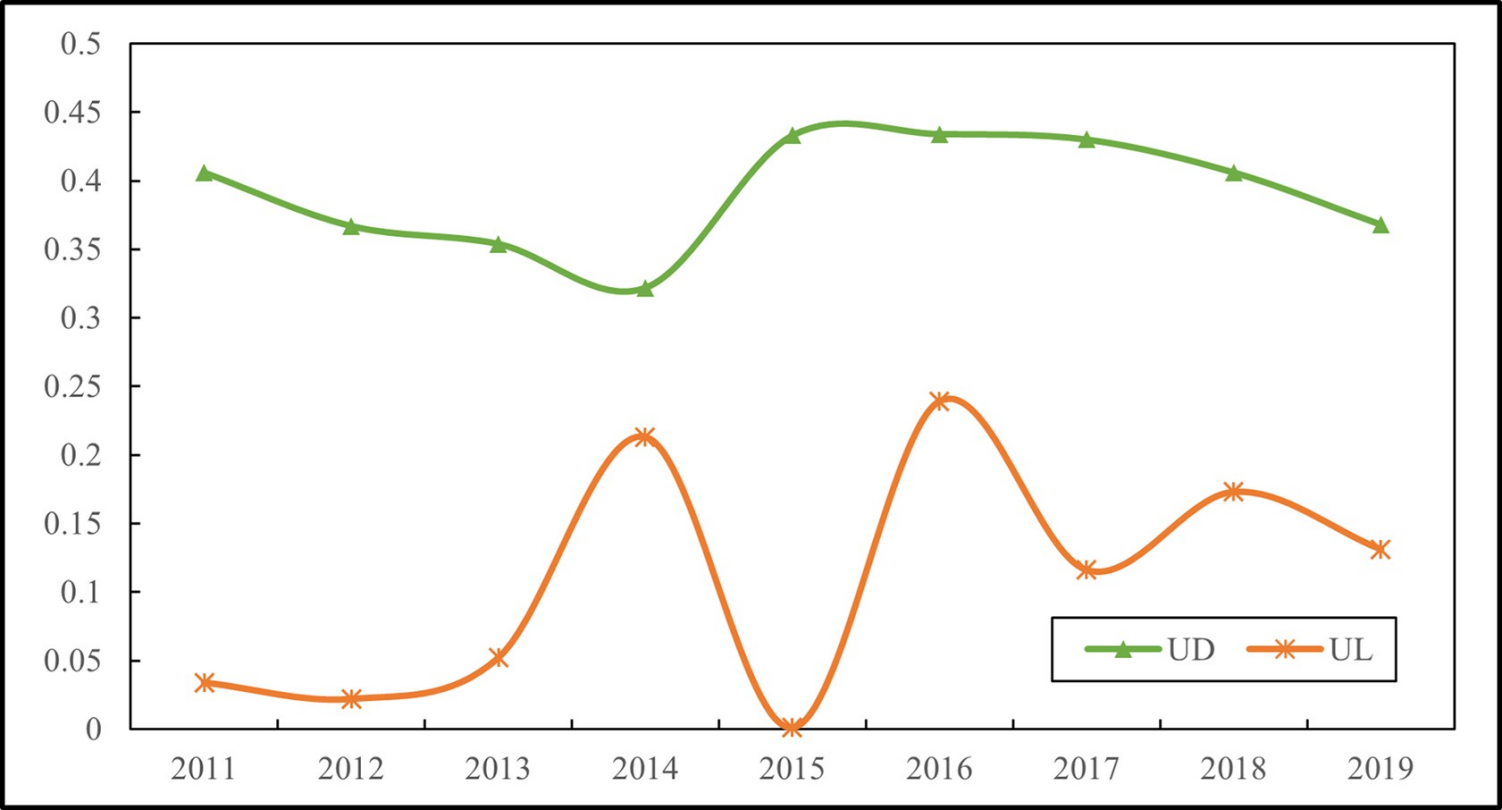
Test of spatial correlation of urban digitalization and low-carbonization.

Fig 2 shows the spatial correlation test for urban digitalization and low-carbonization. The results show that throughout the study period, the GMI values between digitalization and low-carbonization in most cities are significant at the 1% or 5% level and are both positive, demonstrating that the attribute distribution of digitalization and low-carbonization in all cities has a positive spatial correlation and shows a certain agglomeration phenomenon. The GMI for low-carbon development is increasing, indicating a continuous improvement in the spatial spillover effect of the level of low-carbon development among regions. The GMI for urban digitalization shows a downward trend since 2015, indicating that changes in urban digitalization levels have a local agglomeration effect and a decrease in spatial correlation. The possible reason for the above phenomenon is that the central and western regions are developing rapidly under the influence of national strategies, such as the development of the western region and the rise of the central region. Relying on a series of national policies, Chongqing and Hubei have developed strongly. The strong siphoning effect leads to a large loss of human capital in neighboring areas, and the gap between inner cities continues to widen, leading to an increasing decentralization of global spatial autocorrelations.

This paper attempts to explain the spatial distribution law of urban digitalization and low-carbonization. The geographical environment of neighboring regions, resource endowments, trade behavior, and economic decision management have certain similarities, and the clustering of economic activities within spatial units is an important reason for the spatial dependence of the two. Therefore, when analyzing their interactions, it is necessary to take into account the spatial correlations and construct spatial econometric models.

#### Interactive spillover effects of urban digitalization and low-carbonization

Columns (3) to (6) in Table 3 are the estimation results based on the spatial panel simultaneous equation model and GS3LS. To assure the robustness of the results, the spatial weight matrix of geographical distance is used in columns (3) and (4), and the spatial weight matrix of economic-geographical distance is used in columns (5) and (6).

**Table 3:** Estimation results of 3SLS and GS3SLS.

	3SLS		GS3SLS			
			W1		W2	
	(1)	(2)	(3)	(4)	(5)	(6)
VARIABLES	UL	UD	UL	UD	UL	UD
**UL**		0.023***		0.587***		0.372***
		(0.005)		(0.11)		(0.03)
**UD**	0.339***		0.766***		1.145***	
	(0.073)		(0.127)		(0.103)	
W1*UL			1.203***	-0.733***		
			(0.073)	(0.081)		
W1*UD			-0.470***	0.586***		
			(0.085)	(0.034)		
W2*UL					0.106***	-0.074***
					(0.009)	(0.003)
W2*UD					-0.244***	0.168***
					(0.024)	(0.008)
**Other variables**	YES	YES	YES	YES	YES	YES
**Constant**	-0.144	0.001**	-0.080*	0.074**	0.502***	-0.229***
	(0.1249)	(0.050)	(0.006)	(0.034)	(0.043)	(0.019)
**Adj. R** ^**2**^	0.217	0.939	0.070	0.539	0.125	0.702
**Observations**	2,538	2,538	2,538	2,538	2,538	2,538

*** p<0.01

** p<0.05, standard errors in parentheses.

From the point of view of spillover effects, estimated digitalization and low-carbonization variables, including spatial delay conditions under two spatial weight matrices, passed the 1% significance test, suggesting that urban digitalization and low-carbonization had substantial spatial spillover effects. In Eqs (3) and (5), the spatial lag coefficient of low-carbonization (W1*UL) is notably positive, showing that the diffusion effect of low-carbonization has a positive correlation with the increase (decrease) of local low-carbonization levels and the increase (decrease) of low-carbonization levels in neighboring cities. The spatial delay coefficient (W*UD) in digitalization Eqs (4) and (6) is notably positive at the 1% level, demonstrating that digitalization is tightly associated with Chinese cities. Furthermore, two regions with greater rates of digitalization will have a beneficial impact on nearby regions, i.e., a major contribution to improving digitalization in neighboring regions by increasing digitalization in the local region.

From the point of view of the spatial interaction effects, the coefficients of spatial delays (W*UD) in urban digitalization are notably negative in columns 3 and 5, revealing that a drop in surrounding digitalization levels leads to an improvement in low-carbonization local levels. In other words, there is a negative geographical association between neighboring region digitalization and low-carbon localization. The coefficient of the spatial delay term (W*UL) of low carbonization is notably negative in columns 4 and 6, implying that low carbonization has a negative spatial correlation with digitalization in urban areas, i.e., that an increase in the level of low carbonization in neighboring areas can significantly impede the construction of local digitalization.

### Coordinated measures and spatiotemporal pattern analysis for urban digitalization and low-carbonization

#### Timing changes the characteristics of the synergetic advancement of urban digitalization and low-carbonization

We produce the 3D kernel density curve of the coupling coordination degree of urban digitalization and low-carbonization from 2011 to 2019. From the change in the curve’s position, the kernel density curve from 2011 to 2019 gradually moved to the right, showing that urban digitalization and low carbon synergy have a growing upward trend. According to the number of curve peaks, the nuclear density curve was unimodal between 2011 and 2019, indicating that the degree of synergy between urban digitalization and low carbonization was unipolar. From the peak kurtosis of the curve, the curve showed a slight tendency between 2011 and 2019 to increase and then decrease kurtosis, indicating that regional differences in the degree of synergy have declined gradually over the past few years. From 2011 to 2019, the right trawl of the curve is larger than the left trawl, and the right trawl shows a trend of lengthening and thickening, showing that the synergy of high-value regions has improved and the number of cities in high-value regions has increased. Finally, urban digitalization and low-carbonization synergy in different stages of development and polarization have different developmental and polarization characteristics, but overall, it shows continuous improvement in evolutionary characteristics and gradually leads to balanced development trends.

#### Spatial distribution of synergy between urban digitalization and low-carbonization

This article focuses on the synergy of urban digitalization and low-carbonization at five levels: highest, higher, medium, lower, and lowest in 2011, 2014, 2017, and 2019. Between 2011 and 2019, the spatial pattern of the degree of synergy between digitalization and low carbonization in Chinese cities changed dramatically. The level of synergy between urban digitalization and low carbonization is rising all the time, with lowest-level synergy regions steadily vanishing and being replaced by higher-level synergy areas, highest-level synergy areas, and medium-level synergy areas. The spatial structure of the highest-level synergy area gradually extends from the southeast to the northwest. During the study period, higher and highest synergy areas of urban digitalization and lowest carbonization displayed the spatial evolution characteristics of the distribution of "Distributed—Cluster Distribution—Consistency".

Specifically, in 2011, lowest-level and lower-level cities with contiguous distribution and medium-level and highest-level cities with scattered distribution dominated. Highest-level cities such as Beijing, Tianjin, Shanghai, Nanjing, Guangzhou, and Shenzhen started to appear. By 2014, the proportion of lowest-level and lower-level cities in the synergy degree of urban digitalization and carbonization had shrunk. Medium synergy cities show a group development trend and are mainly located in southwest, northeast, and north China, while higher synergy cities show a scattered distribution. Highest-level cities are scattered and distributed in clusters in east and south China, such as Shanghai, Nanjing, Wuxi, Changzhou, Suzhou, Guangzhou, Shenzhen, Zhuhai, and Foshan. In 2015, the lowest- synergy cities were scattered and distributed in sheets. Medium synergy cities were mainly found in the southwest and northwest China. Regions with high and higher levels of coordination are in a decentralized distribution state, while eastern coastal areas are in a group distribution state, such as cities in Shandong, Jiangsu, Zhejiang, Fujian, and Guangdong provinces. In 2019, the proportion of cities with the highest or higher level of synergy increased significantly. The trend of highest-level development in the contiguous areas of eastern and southern China became more obvious. The highest-level regions in northwest central China and northeast China showed a group distribution. However, medium synergy cities were mainly found in the southwest and northwest China. The highest and higher synergy regions were in a decentralized distribution state, while eastern coastal areas were in a group distribution state, such as cities in Shandong, Jiangsu, Zhejiang, Fujian, and Guangdong provinces. Cities with lowest and lower synergy all showed a trend of divergent distribution, such as Songyuan, Mudanjiang, Shangrao, Zaozhuang, Chongzuo, Zhoukou, Zunyi, and Anshun.

### Spatial correlation analysis of synergy between urban digitalization and low-carbonization

#### Overall spatial correlation

Table 4 shows the GMI values of the degree of the synergy between digitalization and low- carbonization in cities. Between 2011 and 2019, all of China’s digitalization and low-carbonization GMI values exceeded 0.015 and 0.102, respectively, and passed the 1% significance test, showing that the degree of digitalization and low-carbonization synergy was significantly positive spatial correlation and demonstrated a strong agglomeration phenomenon in each city during the sampling period. In terms of time, GMI value exhibits a slightly varying trend over the sample period, and in the last five years, the synergy level of urban digitalization and low carbonization has fluctuated relatively steadily around 0.9, demonstrating that the combined synergy level of urban digitalization and low carbonization is an agglomeration phenomenon, but relatively stable. In the future, we should prioritize low synergy levels, regional development gaps, and the coordinated growth of digital and low-carbon cities across the country.

**Table 4:** Global Moran’s I value of synergy degree between urban digitalization and low-carbonization.

	Moran’s I	sd(I)	Z-value	P-value
**2011**	0.096***	0.005	19.366	0.000
**2012**	0.086***	0.005	17.514	0.000
**2013**	0.015***	0.005	3.759	0.000
**2014**	0.095***	0.005	19.309	0.000
**2015**	0.098***	0.005	19.945	0.000
**2016**	0.102***	0.005	20.654	0.000
**2017**	0.085 ***	0.005	17.327	0.000
**2018**	0.092***	0.005	18.703	0.000
**2019**	0.085***	0.005	17.333	0.000

#### Analysis of local spatial correlations

We draw a LISA agglomeration map drawn by matching the attributes and locations of each sample city, which clearly reveals the spatial distributional features of the local spatial autocorrelation.

First, the "high-high (H-H)" type cluster region. In other words, the local cities have a high degree of synergy with their neighbors, indicating a high level of synergy concentration in the region, which has a positive influence relationship. In 2011, the H-H type regions were mainly concentrated in the eastern coastal region, including some cities in Tianjin, Jiangsu, Shanghai, Zhejiang and Fujian provinces. In 2019, the H-H-type was still present in the eastern coastal region, but the clustering area had shrunk somewhat, indicating that the radiative driving effect of regions with high synergy on neighboring regions has not yet played a significant role.

Second, the "high-low (H-L)" type cluster region. In other words, local urban regions have strong synergy, but adjacent cities have poor synergy and negative spatial correlation. In the western and northeastern areas, H-L- type regions are rare and few between. To accomplish reciprocal uplift and mutual advancement between the central city and the surrounding areas, it is required to fully exploit the radiating effects of cities with a high degree of central synergy in this sort of region.

Third, the "low-high (L-H)" type cluster region. In other words, the synergy of the local city is low, while the synergy of the neighboring city is high. The L-H-type region is mainly concentrated in some cities in southwest China. In 2011, Yunfu, Qingyuan, Shaoguan, Heyuan, Shanwei and Chengde were in this type of region. In 2019, the L-H region included nine cities, including Yunfu, Heyuan, Shanwei, Meizhou, and Longyan. This type of regional city was adjacent to an H-H-type agglomeration area, which loses human and financial resources in quantity and is not conducive to local development, thus forming a trough region.

Fourth, the "low-low (L-L)" type agglomeration region. In other words, cities with low synergy with neighboring cities are shown to be agglomeration regions with low synergy and significant positive spatial correlation. The L-L-type regions were concentrated in some western and central cities, with 49 cities in 2011 and 34 cities in 2019. This type of region belongs to the inland regions, which are hindered by transportation and other conditions, the development of innovation in science and technology and human capital is not ideal, the natural environment is poor, and the economic base is weak, limiting the level of synergy improvement.

### Analysis of drivers for synergistic advancement of urban digitalization and low-carbonization

#### Benchmark regression results

The benchmark regression results for the fixed-effects model and the spatial panel data model based on the spatial adjacency matrix are shown in Table 5. Column (1) uses the standard panel regression model. Columns (2) through (5), respectively, employ the models of SAC, SDM SAR, and SEM. According to the results of columns (2), (3), and (5), the spatial lag term ρ is significant, suggesting that the degree of synergy results in essential spatial spillover effects, that is, the synergistic advancement of urban digitalization and low-carbonization is not only influenced by local factors but also by the synergistic advancement level of other cities. The effect is more pronounced in geographically adjacent cities. Therefore, in the context of promoting coordinated regional development, different cities should make full use of their geographical proximity and close economic ties to smooth the flow of factors, carry out extensive and in-depth exchanges and cooperation, promote the sharing of digital technologies and low-carbon scientific research results, and fully play the radiating and driving role of regional central cities.

**Table 5:** Baseline regression: Based on adjacent space weight matrix.

	(1)	(2)	(3)	(4)	(5)
VARIABLES	FE	SAC	SAR	SEM	SDM
**HC**	0.099***	0.094***	0.097***	0.100***	0.078***
	(12.12)	(10.66)	(12.55)	(12.69)	(8.47)
**GC**	0.009**	0.008**	0.008**	0.009**	0.007*
	(2.41)	(2.44)	(2.51)	(2.54)	(1.76)
**OU**	-0.000	0.003	-0.000	0.000	0.001
	(-0.02)	(0.94)	(-0.02)	(0.08)	(0.30)
**TI**	0.026***	0.032***	0.026***	0.027***	0.029***
	(6.37)	(7.22)	(6.55)	(6.66)	(5.24)
**ED**	0.030***	0.031***	0.028***	0.029***	0.031***
	(5.68)	(5.10)	(5.68)	(5.78)	(4.64)
**IS**	-0.004**	-0.004**	-0.004**	-0.004**	-0.004**
	(-2.16)	(-2.23)	(-2.24)	(-2.28)	(-1.98)
**US**	0.011***	0.010***	0.011***	0.011***	0.009***
	(3.58)	(3.96)	(3.80)	(3.85)	(3.11)
**CF**	0.001***	0.001***	0.001***	0.001***	0.001***
	(3.82)	(5.66)	(4.37)	(4.54)	(3.64)
**ρ**		0.647***	0.053*		0.046*
		(12.42)	(1.96)		(1.66)
**λ**		0.603***		0.062**	
		(16.68)		(2.16)	
**Constant**	-0.840***				
	(-8.83)				
**Year Effect**	Yes	Yes	Yes	Yes	Yes
**City Effect**	Yes	Yes	Yes	Yes	Yes
**Log-lik**		5998.4053	5972.8043	5973.3395	6024.9403
**R-squared**	0.704	0.238	0.260	0.284	0.225
**Observations**	2,538	2,538	2,538	2,538	2,538

Note

*** p<0.01

** p<0.05

* p<0.1, t(z)-statistics in parentheses.

From the perspective of impact factors, economic development, human capital, and technological innovation are critical driving forces for the synergetic promotion of urban digitalization and low-carbonization. Specifically, in terms of internal driving forces, both economic development and human capital pass the significance test at 1%, demonstrating that human capital and economic development are vital internal driving forces for the synergetic promotion of urban digitalization and low-carbonization. The level of industrial structure upgrading is significantly negative at 5%, showing that a low level of industrial structure upgrading or an excessively high level of industrialization in a city is not conducive to the synergistic advancement of urban digitalization and low-carbon development, and the "clean", "light" and "service" development of the industrial structure should be promoted. In terms of external drivers, Government capacity is highly positive in all models, demonstrating that government capacity is an essential external driver for the synchronized growth of urban digitalization and low-carbon development. The government’s function and involvement in planning and driving the transformation of cities to digital and low-carbon growth should be expanded in the future. The computed coefficients for the degree of openness to the outside world are not significant, indicating that the function of overall openness to the outside world in the synergistic process of urban digitalization and low-carbonization has not yet been completely realized. In the future, we should accelerate the change of the foreign trade growth pattern, strengthen the international division of labor, and encourage high-quality foreign trade expansion. At the level of 1%, technological innovation passes the significance test, suggesting that increasing the level of urban technological innovation can greatly increase the synergistic advancement degree of digitalization and low-carbonization. In terms of control variables, both city size and infrastructure level are significantly positive at 1%, implying that urban infrastructure improvement and economies of scale are the primary drivers of modern urban development, which can provide new impetus to the integration and collaboration of urban digital construction and urban low-carbon development.

#### Robustness test

Considering that spatial panel models specified by a single spatial weight matrix can produce contingency regression results. Therefore, in this paper, we choose the inverse distance space weight matrix for further testing. Table 6 shows the robustness test results. The estimates of the eigenvalues and significance for the main variables of the spatial panel model with different spatial weight matrices agree with those in Table 5, but the magnitude and significance of the coefficients differ slightly, indicating that the conclusions of this paper are relatively robust.

**Table 6:** Robustness test: Based on inverse distance space weight matrix.

	(1)	(2)	(3)	(4)
VARIABLES	SAC	SAR	SEM	SDM
**HC**	0.101***	0.095***	0.098***	0.080***
	(12.88)	(12.00)	(12.32)	(8.60)
**GC**	0.009***	0.009**	0.009**	0.009**
	(2.81)	(2.52)	(2.56)	(2.49)
**OU**	-0.001	-0.000	0.000	0.001
	(-0.23)	(-0.06)	(0.07)	(0.26)
**TI**	0.028***	0.025***	0.027***	0.037***
	(7.20)	(6.48)	(6.74)	(7.29)
**ED**	0.026***	0.027***	0.030***	0.031***
	(5.44)	(5.30)	(5.67)	(4.57)
**IS**	-0.004**	-0.004**	-0.004**	-0.004**
	(-2.54)	(-2.17)	(-2.32)	(-2.09)
**US**	0.011***	0.011***	0.011***	0.008***
	(4.24)	(3.83)	(3.94)	(3.05)
**CF**	0.001***	0.001***	0.001***	0.001***
	(4.33)	(4.34)	(4.68)	(2.96)
**ρ**	0.545***	0.298**		0.132
	(8.52)	(2.27)		(0.85)
**λ**	0.544***		0.415***	
	(9.96)		(3.13)	
**Constant**				
**Year Effect**	Yes	Yes	Yes	Yes
**City Effect**	Yes	Yes	Yes	Yes
**Log-lik**	5983.1428	5973.3853	5975.1144	5974.7926
**R-squared**	0.201	0.308	0.290	0.051
**Observations**	2,538	2,538	2,538	2,538

Note

*** p<0.01

** p<0.05

* p<0.1, t(Z)-statistics in parentheses.

## Discussion

Strengthening theoretical understanding and empirical research on the synergistic progress of urban digitalization and low-carbonization is a new understanding to promote the deep integration of urban digitalization and low-carbonization in the current era of the global digitalization and low-carbonization revolution. Numerous academics have focused on the connection between urban digitalization and low-carbonization [33, 93, 94]. The findings of this study further support the findings of earlier studies [24, 47] that urban digitalization promotes low-carbonization. Unlike previous studies, this paper investigates the two-way connection between urban digitalization and low-carbonization from the viewpoint of endogenous interaction, evaluates the spatiotemporal heterogeneity, spatial correlation, and influences of the synchronized development of urban digitalization and low-carbonization, and more comprehensively and scientifically understands the coordination and interaction mechanism, evolution law and driving factors of urban digitalization and low-carbonization from the regional systematic and comprehensive perspective. Furthermore, based on the examination of a linked coordination model between geographical and physical spatial viewpoints, this paper proposes a new paradigm and viewpoint extension for the study of urban digitalization and low-carbonization. The study of the interaction between urban digitalization and low-carbonization reported in this paper demonstrates that both digitalization and low-carbonization have a beneficial geographical spillover impact on nearby cities. As a result, the government should implement categorization rules based on regional differences, as well as strengthen regional cooperative governance models [95, 96]. On the one hand, we should focus on developed cities in eastern China and low-carbon piloting cities in central and western China, promote the opening up and sharing of low-carbon scientific research achievements, and speed up the low-carbon transition in backward regions through demonstration effects. On the other hand, measures should be taken based on local conditions and categories to gradually close the gap in regional digital development and propel the region as a whole to the stage of superior digital advancement, depending on the economic development level and resource endowment of each region.

In addition, the study discovered negative spatial interaction effects in nearby cities for both digitalization and low-carbonization. The obvious spillover impact of urban digitalization and low-carbonization is the most plausible explanation [97, 98]. On the one hand, cities that experience rapid digital growth can attract resources such as expertise and low-carbon technology to the region, impeding low-carbon development in surrounding cities. On the other hand, cities with a high level of low-carbon development, which rely on advantages such as a decent ecological environment and industrial structure, create a siphoning effect on the capital, talent, and additional resources of neighboring cities [99], thus negatively affecting their digital construction. Therefore, when formulating relevant policies, the government should focus on policy coordination among different cities, avoid ineffective and inefficient competition among cities, and minimize the negative impact of digitalization and low-carbon development on neighboring cities.

This study discovered that, while the synergistic advancement degree between urban digitalization and low-carbonization is continuously improving, the overall level is low and spatial heterogeneity is significant by analyzing the spatiotemporal evolution characteristics of the cooperation degree between urban digitalization and low-carbonization. Combined with practical development, we should first do an adequate task of integrating ecological preservation and digital construction [100, 101], and encourage the conservation of energy, emission reductions, and sustainable growth in areas where energy consumption is high, such as the 5G network, big data centers, and industrial networks. Second, we should allocate digital construction in a rational way based on regional resource endowments and locations, and promote coordination and linkages between eastern, central, and western regions. We need to foster a fresh ecosystem for urban development in science and technology innovation, industrial development, urban governance, and social services, raise the level of green and low-carbon urban development, and promote coordinated regional development. Third, cities in the central and western regions should learn from the development experience of the eastern regions and adopt a diversified development approach that takes into account local conditions and times. At the same time, appropriate policies and measures should be formulated on talent development, industrial restructuring, and innovation-driven development to further narrow regional disparities in the synergistic advancement of digitalization and low-carbonization.

However, there are several limitations to this study. For starters, this study simply looks at the overall geographical interaction impacts of urban digitalization and low-carbonization without delving into the breakdown of spatial spillover effects and the varied features of different locations. Second, this study does not further explore the internal transmission mechanisms of coordinated urban digitalization and low-carbonization. Therefore, future studies can further explore the following aspects. First, further decompose and test the spatial spillover effects of the interaction between urban digitalization and low-carbonization in both temporal and spatial dimensions, thus providing concrete guidance for improving the spatial allocation efficiency of digital and low-carbon resources. Second, the inclusion of mediating variables in the model clarifies the mechanism of occurrence and the path of implementation of the interplay between urban digitalization and low-carbonization, thus providing an experience for promoting regional digitalization construction and improving urban low-carbon development.

## Conclusions

Based on the theoretical framework construction of the internal mechanisms of mutual boosting of urban digitalization and low-carbonization, as well as the key factors of synergistic advancement, this paper selects 282 cities in China from 2011 to 2019 as a sample for designing spatial simultaneous equation models and investigates the interplay between urban digitalization and low-carbonization using the GS3SLS method. Then, utilizing 3D kernel density, spatial statistics, and panel spatial econometric models, we examine the spatiotemporal evolution features of urban digitalization and low-carbonization, as well as the impact factor of synergistic advancement. The main conclusions are: First, there is a mutually reinforcing endogenous interaction between urban digitalization and low-carbonization, and this interaction pattern is subject to a certain geographical proximity. Second, over the research period, the coordination of urban digitalization and low-carbonization increased, indicating a tendency for unipolar growth. The synergy degree between urban digitalization and low-carbonization has a significant positive spatial correlation, and the types of H-H and L-L aggregates decrease over time and tend to develop in a spatially balanced manner. Third, the regional synergy of urban digitalization and low-carbonization produces a large geographical spillover impact. Fourth, the degree of economic growth, industrial structure, and human capital accumulation are significant internal elements for the synergistic advancement of urban digitalization and low-carbonization promotion. The primary external variables for the synergistic advancement of urban digitalization and low-carbon development are government capability and technical innovation, but the uplifting impact of more openness to the outside world on the coordinated development of both is less evident.
